# Fate of nanoparticles in the central nervous system after intrathecal injection in healthy mice

**DOI:** 10.1038/s41598-019-49028-w

**Published:** 2019-08-29

**Authors:** K. T. Householder, S. Dharmaraj, D. I. Sandberg, R. J. Wechsler-Reya, R. W. Sirianni

**Affiliations:** 1 Vivian L. Smith Department of Neurosurgery, McGovern Medical School, University of Texas Health Science Center at Houston, 6431 Fannin St, Houston, TX 77030 USA; 2 Barrow Brain Tumor Research Center, Barrow Neurological Institute, 350W. Thomas Rd, Phoenix, AZ 85013 USA; 3 School of Biological and Health Systems Engineering, Ira A. Fulton Schools of Engineering, Arizona State University, P.O Box 879709, Tempe, AZ 85287 USA; 4 Departments of Pediatric Surgery and Neurosurgery, McGovern Medical School, University of Texas Health Science Center at Houston, 6431 Fannin St, MSB 5.144, Houston, TX 77030 USA; 5 NCI-Designated Cancer Center, Sanford-Burnham Medical Research Institute, 10901N Torrey Pines Rd, La Jolla, CA 92037 USA

**Keywords:** Permeation and transport, Nanoparticles

## Abstract

Cerebrospinal fluid (CSF) is produced in the cerebral ventricles and circulates within the subarachnoid space (SAS) of the brain and spinal cord, where it exchanges with interstitial fluid of the parenchyma. The access of CSF to the entire central nervous system (CNS) makes it an attractive medium for drug delivery. However, few intrathecal (IT) therapies have reached the clinic due, in part, to limited distribution and rapid clearance. Given the success of nanoparticle (NP) carriers in prolonging circulation and improving delivery of systemically administered agents, we sought to evaluate the distribution of IT injected NPs within the CNS. We administered fluorescent, 100 nm PEGylated-NPs into the cisterna magna of healthy mice and studied their distribution along the brain and spinal cord. Our data demonstrate that NPs are capable of distributing rapidly through the SAS along the entire neuraxis with reproducible, anatomically defined patterns of delivery. NPs were well retained within the leptomeninges for over 3 weeks, showing preference for ventral surfaces and minimal penetration into the CNS parenchyma. Clearance of NPs occurred across the cribriform plate into the nasal mucosa, with a small fraction of NPs localizing with nerve roots exiting the spinal column. Larger 10 µm particles were also capable of moving through the SAS but did not achieve as widespread distribution. These studies demonstrate the ability of NPs to achieve widespread delivery along the neuraxis and highlight IT administration as a potentially significant route of administration for delivery of nanomedicine to the subarachnoid space.

## Introduction

Intrathecal drug administration, i.e. the delivery of active agents directly into the CSF, has gained renewed attention as an approach that could circumvent systemic barriers to CNS drug delivery. IT administration achieves high concentrations of drug in the CSF that bathes the brain and spinal cord with minimal systemic exposure^1–6^. However, poor drug solubility, inadequate pharmacokinetics, limited tissue distribution, and neurotoxicity remain significant obstacles for most IT administered agents^7–12^. Ultimately, few drugs possess biophysical properties that are compatible with delivery via this route. Hydrophilic compounds typically distribute rapidly through the SAS but are cleared quickly with CSF turnover, requiring frequent dosing to maintain drug levels^13–17^. Hydrophobic/lipophilic compounds are often limited by poor solubility. Additionally, partitioning of small molecules into lipophilic cell membranes and binding to extracellular matrix proteins severely restricts their distribution away from the site of injection, making the approach unsuitable for treating diseases that affect non-focal regions of the CNS^15,18–20^.

Encapsulation of therapeutic molecules within polymeric or liposomal NPs is a strategy that has been utilized extensively to overcome biophysical and pharmacokinetic limitations of systemically administered agents^21–26^. The versatility of NP systems allows for a wide range of compounds to be effectively encapsulated and released over prolonged periods of time^23^. Importantly, intravenously administered NPs have been engineered to traverse a variety of biological barriers through optimization of properties such as size and surface features.

We hypothesized that IT administered NPs would be effectively distributed through the SAS by the convective forces of the CSF, enabling them to reach CSF-exposed surfaces of the brain and spinal cord. To test this hypothesis, we administered fluorescently labeled, non-degradable polystyrene NPs to healthy mice via direct injection into the cisterna magna. This model NP platform was selected for several reasons, including monodisperse size, stable internal fluorescent labeling, and surface chemistry that is amenable to covalent modification. NP delivery was evaluated by a number of approaches to characterize distribution both along the neuraxis and within the SAS. These studies show the potential of NPs to achieve widespread delivery along the neuraxis after IT administration and serve as a mechanistic foundation to guide the future design of NPs for IT delivery of therapeutics to treat diseases of the CNS.

## Results

### FNP PEGylation

Successful PEGylation of fluorescent, polystyrene nanoparticles (FNPs) via EDC chemistry was confirmed by the appearance of a peak at 3.6 in the pegylated FNPs when analyzed by ^1^H NMR (SI Fig. 1). Additionally, dynamic light scattering showed the expected increase in both average diameter and zeta potential. After PEGylation, the 100 nm FNP diameter increased to 122 ± 8.17 nm and the zeta potential increased from −45 ± 5.4 to −14 ± 2.2 mV. FNPs were confirmed to be stable in protein rich environments, since DLS measurements of FNPs incubated in 10% FBS at 37 °C showed no significant changes in size, PDI or zeta potential over one week (SI Fig. 2).

### *In Vivo* FNP Tracking

**Figure 1:**
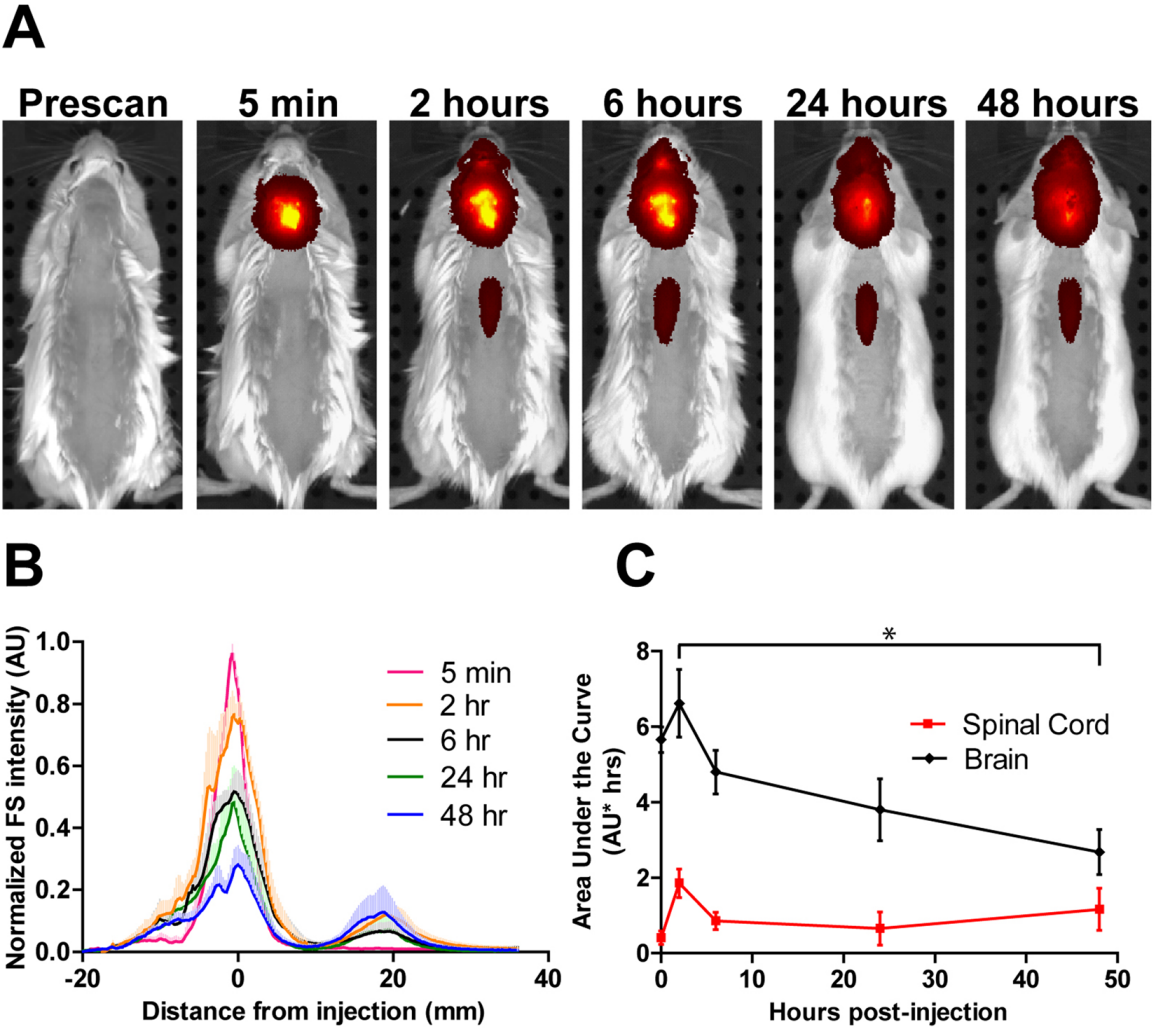
Semi-quantitative IVIS tracking of FNP distribution after IT administration in intact healthy mice. FNPs can be observed with semi-quantitative measurement over the brain and spinal cord. (**A**) Representative images show the spread of FNPs away from the cisterna magna over time. FNPs were detectable along the spinal cord by 2 hours but only along the mid of the back unobstructed by the extra muscle of the forelimbs and hindlimbs. (**B**) A graph of FNP intensity along a line profile from nose to tail demonstrates a relative decrease in total signal along the dorsal brain with a slight increase in signal along the dorsal spine over time. (**C**) AUCs calculated from the brain signal (−20 to 10 mm) and spinal signal (10 to 40 mm) showed significant clearance of FNPs from the brain region from 2 to 48 hours (p < 0.05, 1-way ANOVA with Tukey’s multiple comparison test), while the FNP intensity along the spinal cord did not significantly change over the 48 hours. Mean + SD shown in B to help keep lines visible. AUC points show mean ± SD.

To evaluate broad patterns of NP distribute following intracisternal injection, FNP distribution was monitored semi-quantitatively *in vivo* in healthy mice over 2 days using an IVIS imaging system. Due to the limitations of fluorescence penetration, IVIS imaging in live mice could only reliably detect the FNP signal along a short segment of the spinal column in the middle of the back, where the signal was not obstructed by the musculature of the shoulders and hindlimbs. At 5 min post-injection, the FNP signal was detected at the injection site only (Fig. 1). By 2 hours post-injection, the FNP signal was detected over the entire brain region and along the spinal cord, although the strongest signal remained focused at the injection site. From 2 to 48 hours post-injection, a small degree of redistribution of FNP signal from the brain to the spinal cord was observed. The brain AUC significantly decreased from 6.6 at 2 hrs to 2.7 AU*hrs by 48 hrs (p = 0.0098, Fig. 1C), with the greatest clearance observed from 2 to 6 hrs (6.6 to 4.8 AU*hrs). Although there was a modest decrease in FNP AUC along the spinal cord from 2 to 6 hrs (1.9 to 0.86 AU*hrs), the spinal signal AUC increased to 1.2 AU*hrs by 48 hours.

### *Ex Vivo* Analysis of FNP Spinal Column Distribution

**Figure 2:**
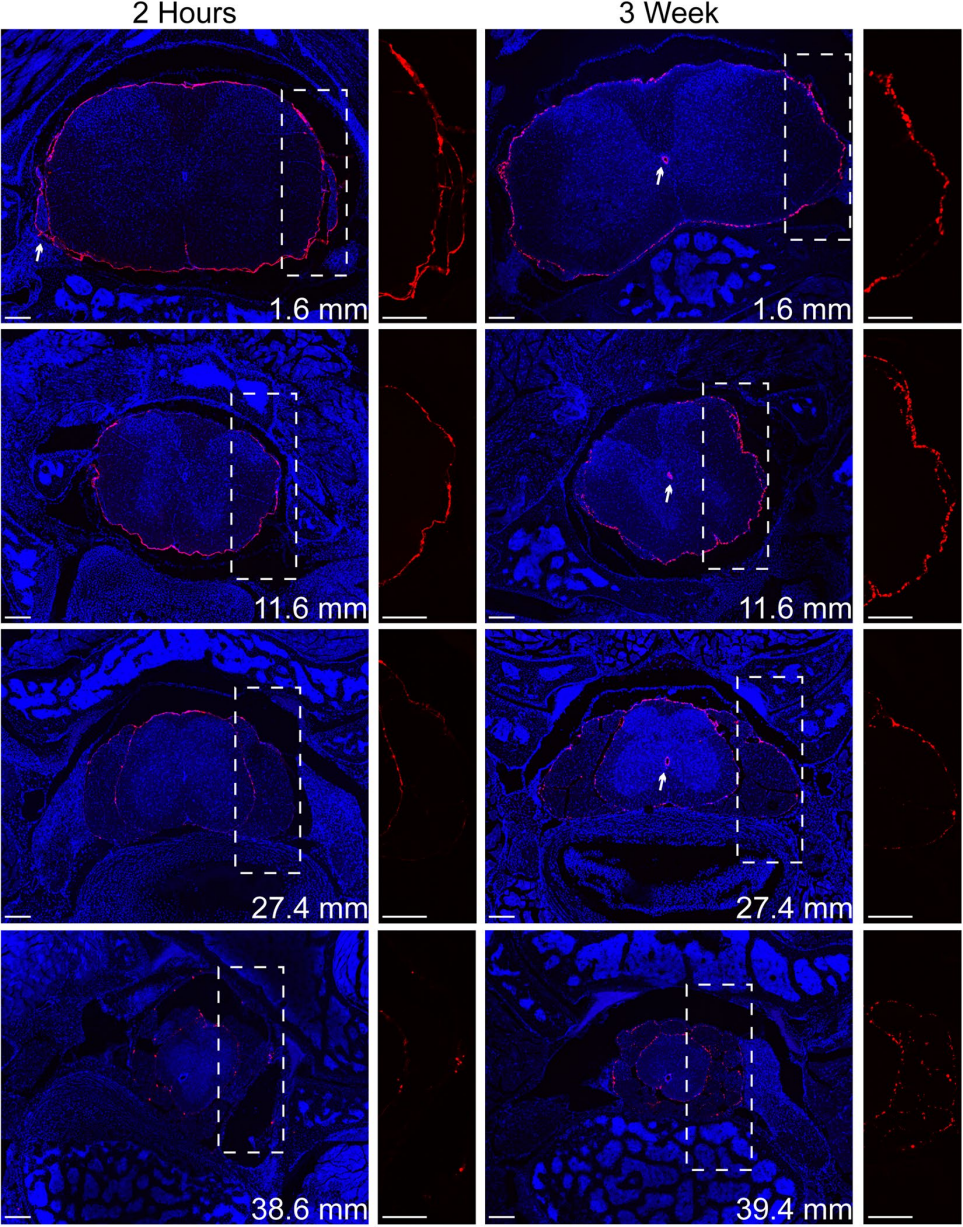
Representative confocal images of FNP distribution along the spinal cord at 2 hours and 3 weeks. FNPs (red) distributed to the sacral regions of the spinal column within 2 hours and remained for over 3 weeks. FNPs could be observed following the meninges around spinal nerve roots (2 hr arrow), which may represent a clearance route for FNPs from the CNS. Even at later time points, the FNPs were mainly confined to meninges with minimal evidence for parenchymal penetration. FNPs appeared more punctate at later time points and could frequently be found within the central canal (3 wk arrows). The location of the slice along the spinal column is shown for each slice in the lower right. Zoomed in image of only FNP signal within the dashed box shown to the right of each tissue slice. Equivalent linear adjustments were made to the FNP signal in each image to enable better visualization. Cell nuclei (DAPI) are shown in blue. Scale bar = 200 µm.

**Figure 3:**
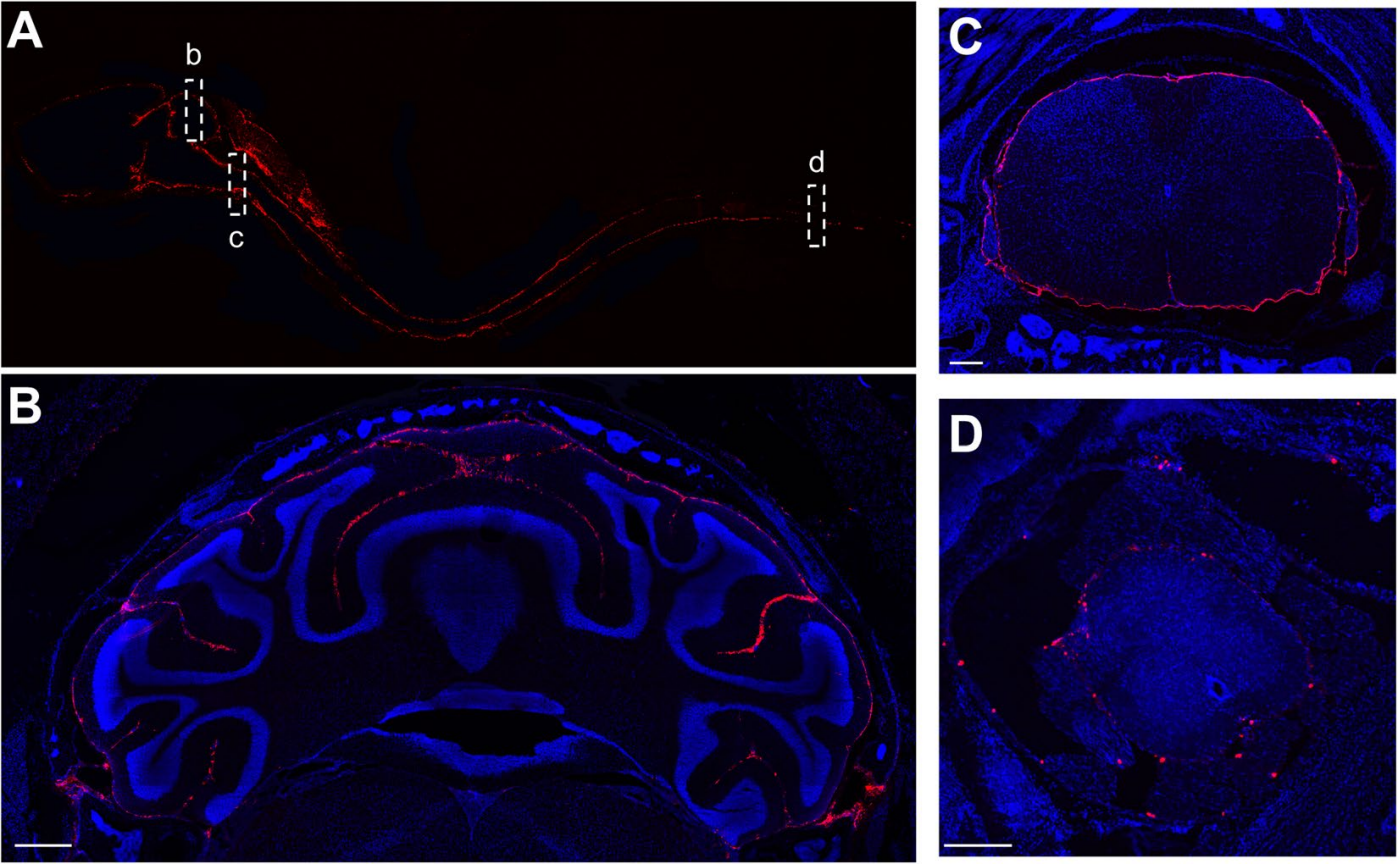
FNPs achieve widespread delivery through the SAS within 2 hours of IT administration. (**A**) Sagittal view of the entire neuraxis showing distribution of FNPs (red) from the olfactory bulb to the sacral spinal cord. (**B**) FNPs were able to access nearly all CSF exposed tissue, including into the sulci of the cerebellum. Along the spinal column, FNPs distributed around the entire circumference of the spinal cord all the way from the cervical (**C**) to the sacral (**D**) regions. Scale bar in B = 500 µm and in C&D = 200 µm.

Transverse tissue slices, taken from known distances along the spinal column, were imaged by confocal microscopy to visualize FNP distribution within the SAS around and along the spinal cord in decalcified samples. IVIS imaging of a complete neuraxis before and after decalcification was examined to confirm the decalcification process did not produce a loss or redistribution of FNP signal (SI Fig. 3). Although the fluorescence signal was observed to increase after decalcification, presumably because the reduction of signal attenuation by bone, the spatial distribution of fluorescent signal along the neuraxis remained consistent before and after decalcification. In a subset of animals, we removed the bone and meninges prior to imaging. Removal of the meninges resulted in a loss of nearly all detectable FNP signal (data not shown), providing evidence that the FNPs are retained within the SAS. Although it is difficult to visualize the boundary between the pia matter and the parenchyma, we did not observe evidence for significant FNP penetration into the spinal cord parenchyma from the CSF. FNPs were observed to traverse the length of the spinal cord within 2 hours of injection, including sacral regions. FNP signal encompassed the entire circumference of the spinal cord (Figs 2, 3 and SI Fig. 4). FNPs were reliably localized with exiting nerve roots (Figs 2 and 3C). We consistently observed a small population of FNPs within the central canal at all time points except at 2 hours, especially in cervical and thoracic spinal sections. Three weeks after injection, the FNPs were still readily detected along the entire length of the spinal column, although they appeared more punctate with slightly larger foci compared to earlier time points (Fig. 2). To test if the rapid distribution of the FNPs to the sacral spinal cord was size dependent, we examined the distribution of 10 µm non-PEGylated FluoSphere microparticles (MPs) within the spinal cord SAS. The MPs could be found in the sacral region within 2 hours, albeit very infrequently (3 total in 54 slices observed, SI Fig. 5).

**Figure 4:**
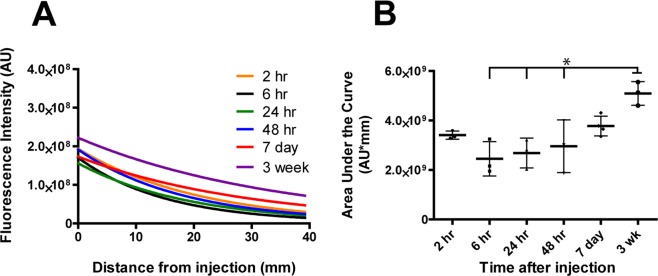
Quantification of FNP intensity along the spinal column following IT injection. (**A**) Exponential curve fits of FNP intensity along the spinal column showed relatively constant decay in FNP signal moving caudally down the spinal cord at all time points. (**B**) AUC of the curve fits for individual mice at each time point (n = 3/time point) shows FNP delivery to the spinal cord trended upwards over time and was significantly higher at 3 weeks compared to 6, 24 and 48 hours (p < 0.05, 1-way ANOVA with Bonferroni’s Multiple Comparison Test). Mean ± SD shown.

Quantification of FNP intensity with ImageJ revealed a gradual exponential decay in fluorescent signal moving caudal from the injection site towards the sacral spinal section (Fig. 4 and SI Fig. 6). The general trends observed from quantification of tissue slices were in agreement with the semi-quantitative measurements generated by IVIS imaging. For the first 48 hours and even at 1 week, the total FNP delivery (AUC) to the spinal column did not significantly change (Fig. 3B). We did observe a similar trend as IVIS with a decrease in AUC from 2 to 6 hours (3.4E9 to 2.4E9 AU), followed by a slight increase in delivery from 24 to 48 hours (2.7E9 to 3.0E9 AU). FNP concentration along the spinal cord continued to trend upwards from 48 hours to 1 week (3.0E9 to 3.8E9 AU) and was significantly higher at 3 weeks post-injection (5.1E9 AU) compared to 6, 24 and 48 hours post-injection (p < 0.05).

### *Ex Vivo* Brain Distribution

**Figure 5:**
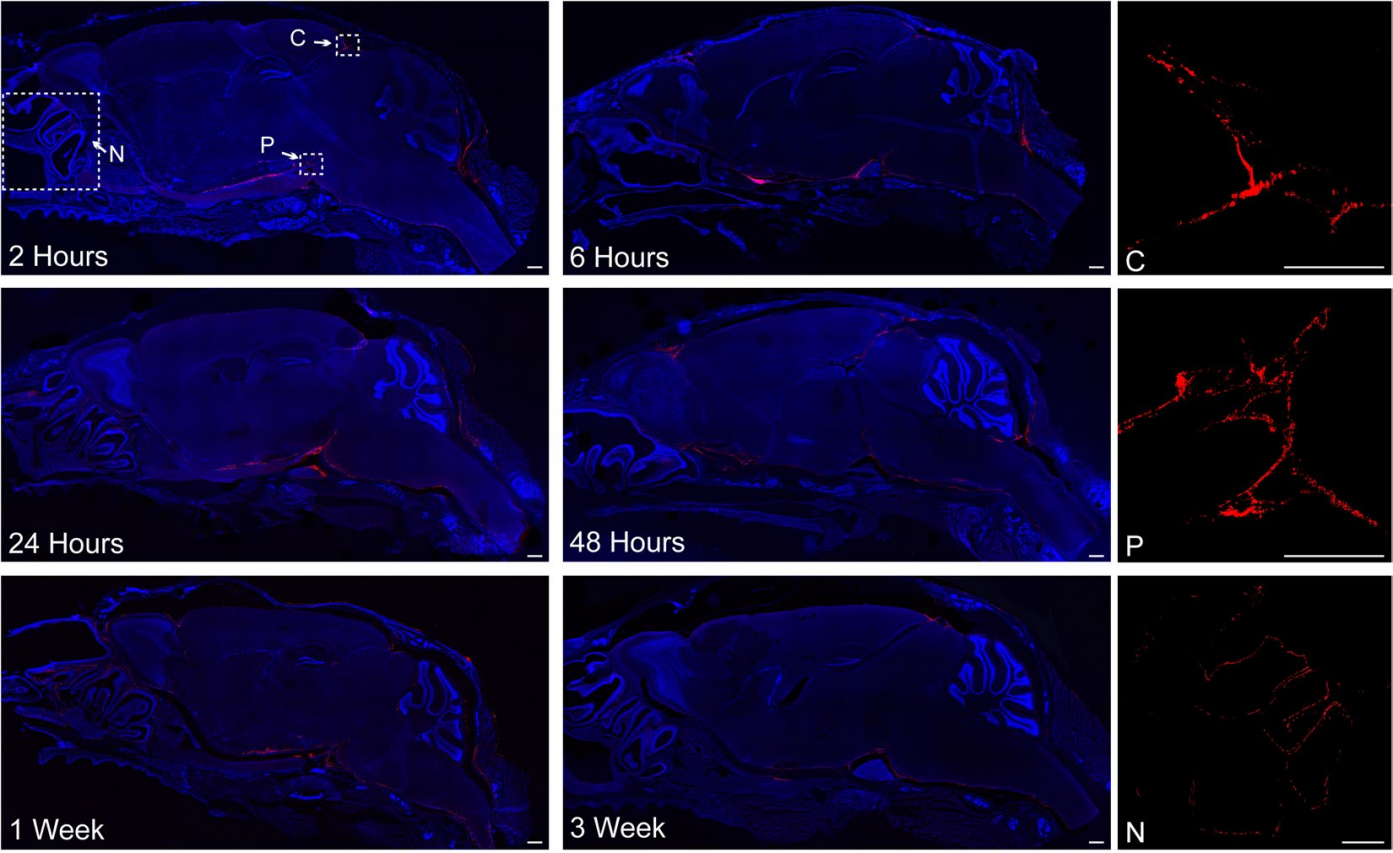
Representative confocal images of FNP distribution around the brain. FNPs (red) were distributed around the entire brain and could be seen following the meninges into the sulci of the cerebellum. There was a strong preference for ventral distribution and consistently high delivery to the supracerebellar cistern (**C**) and pituitary recess (P). Clearance of FNPs across the cribriform plate into the nasal mucosa (N) was seen at all time points. Qualitatively, a general decrease in total FNP intensity was observed over time, consistent with IVIS data. Linear corrections were applied equally across images to better show FNP distribution. Cell nuclei (DAPI) are in blue to show the gross anatomy of the brain. Images without DAPI, to better see FNP signal, can be found in the Supplementary data. Scale bar = 500 µm.

**Figure 6:**
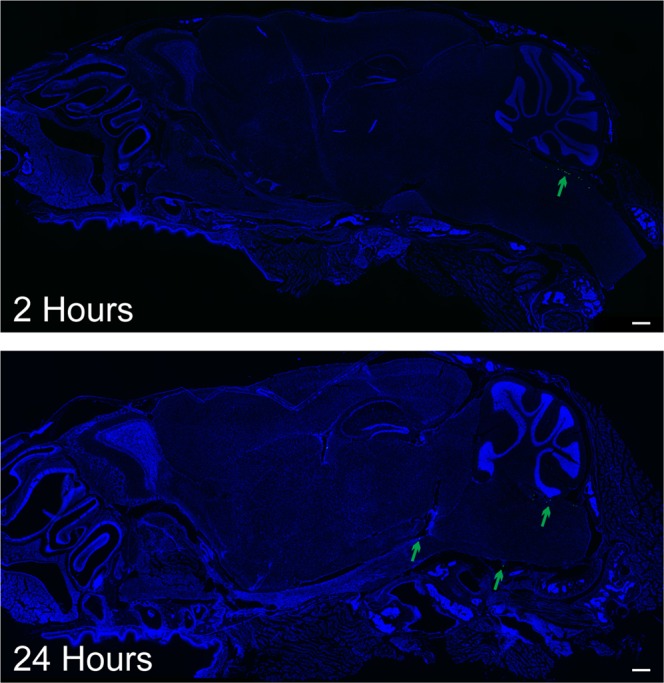
Representative confocal images of MP distribution around the brain. MPs (green) exhibited significantly less mobility around the brain compared to FNPs. Most MPs remained near the injection site, with a few moving along the ventral side of the brain. No MPs were found along the dorsal brain. Cell nuclei (DAPI) are in blue to show the gross anatomy of the brain. Scale bar = 500 µm.

To visualize the anterior/posterior distribution of FNPs around the complex geometries of the brain, the brain was sliced in sagittal sections (Fig. 5 and SI Fig. 7). Consistent with our observations along the spinal cord, we did not find evidence of FNP penetration across the pia mater into the brain parenchyma. In general, FNPs distributed throughout the entire SAS to reach all CSF exposed surfaces of the brain. FNPs were observed to follow the meninges into the sulci of the cerebellum (Fig. 3B) and were also observed along the trigeminal nerve (Fig. 5). In all mice, we observed the greatest concentrations of FNPs within concave structures around the brain, particularly within the supracerebellar cistern between the cortex and cerebellum on the dorsal side of the brain and the pituitary recess anterior of the pons on the ventral side of the brain. FNP delivery was consistently more concentrated along ventral surfaces of the brain compared to dorsal surfaces, and FNP signal was especially sparse along the dorsal prefrontal cortex region at all time points examined. To test whether the ventral preference was an artifact of the mouse lying in the prone position after injection, we evaluated FNP distribution in 3 mice that were maintained in a supine position for 2 hours post-injection and sacrificed immediately thereafter. Recovery position did not produce any identifiable changes to the FNP distribution, and delivery to the prefrontal cortex region remained sparse (SI Fig. 8). The distribution of 10 µm MPs within the SAS of the brain was more limited than FNPs. MPs did not distribute into the sulci of the cerebellum and their delivery to ventral surfaces of the brain was even more limited than FNPs (Fig. 6).

### CNS Clearance

**Figure 7:**
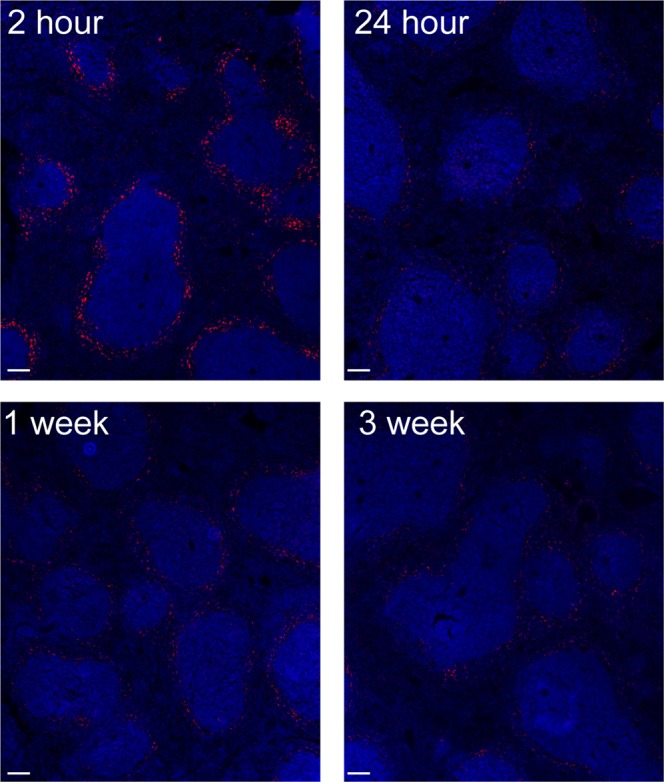
Representative confocal image of FNP distribution to the spleen over time. FNPs (red) appeared to be localized around the white pulp, with the greatest signal observed at 2 hours. Cell nuclei (DAPI) shown in blue. Scale bar = 100 µm.

We observed evidence for FNP transport across the cribriform plate into the nasal sinuses at all time points (Figs 5 and SI Fig. 6). To test whether IT administered FNPs were cleared into peripheral compartments, we examined the liver and spleen with confocal microscopy. There was no observable FNP signal above background in the liver at any of the time points (data not shown). FNPs were detectable within the spleen at all time points, with the greatest concentration of FNPs seen at 2 hours after injection (Fig. 7). Anatomically, FNPs appeared to be consolidated around the white pulps of the spleen.

## Discussion

CSF is produced by the choroid plexus, after which it moves through the ventricles, where it distributes down the spinal cord and around the brain through the SAS^27,28^. The SAS exists between the arachnoid mater and pia mater around the brain and spinal cord. Thin, collagen trabeculae extend between the arachnoid mater and pia mater, generating a mesh-like network within the SAS through which the CSF moves. Although the overall magnitude of a bulk and net directional flow of CSF are debated, it is generally accepted that CSF moves in a oscillatory fashion due to the rhythmic forces generated by the cardiac and respiratory cycles^29–32^. Most therapies of interest for neurological disease achieve poor CSF distribution, and the use of IT administration in the clinic has been mainly limited to analgesics and a few water-soluble molecules. Hydrophilic molecules are known to distribute and clear rapidly as CSF turns over. SPECT studies by Wolf *et al*. suggest that the limited tissue distribution of hydrophobic molecules is due to binding of molecules to cellular and extracellular components of the leptomeninges and SAS, thus highlighting biophysical considerations that could limit the movement of IT administered molecules^15^. Limited distribution is advantageous for the translation of analgesics, where local delivery avoids possible detrimental respiratory side effects. However, limited distribution is not an advantage for lipophilic drugs that need to reach larger tissue volumes, such as chemotherapies.

Nanoparticles have been administered IT previously^20,33–35^. Dengler *et al*. found 230 nm, non-degradable, silica-lipid NPs resided within the meninges for at least 8 weeks after lumbar injection. Multiple reports have shown effective local nano/micro-particle mediated sustained delivery for the treatment of spinal cord injury^36–38^. Shyam *et al*. and Hagihara *et al*. both demonstrated the potential of NPs to transfect cells across multiple CNS regions after IT administration, and Kitamura *et al*. demonstrated that chemotherapy-loaded liposomes could prolong survival in a rat model of meningeal gliomatosis. While these studies establish the potential significance of the IT delivery route, little is known about the spatiotemporal distribution of NPs within the SAS. Thus, the objective of this study was to thoroughly characterize the distribution of solid NPs within the CNS following IT administration.

Injection of both 100 nm FNPs and 10 µm MPs into the cisterna magna was well tolerated in all mice. FNPs were observed to distribute through the meninges along the entire neuraxis, including into the sulci of the brain and along nerve bundles (trigeminal, optic and exiting spinal nerve roots). Distribution of FNPs to the sacral portion of the spinal column within 2 hours post administration is too rapid for Fickian diffusion alone, highlighting a key role for convective forces. Movement of the FNPs in all directions from the cisterna magna supports a model of CSF mixing as opposed to strictly directional laminar CSF flow^29–31,39^.

Recent studies highlight the significance of CSF mixing within the SAS of the spinal cord, with an expectation of decreased oscillation moving caudally along the spinal column^32,40,41^. In the current study, we observed that, while FNPs accessed distal regions of the spinal cord, a gradient of FNP concentration existed in the rostral-caudal direction. This gradient could represent an equilibrium established by decreasing oscillatory forces. The FNP signal became more punctate at 3 weeks, which could be indicative of FNP entrapment in microanatomy or aggregation over time. Based on prior works by Wolf *et al*. and Papisov *et al*., a moderate, exponential FNP gradient along the spinal column is consistent with the distribution of hydrophilic, low-binding small molecules injected intracisternally or intraventricularly^14,15^.

FNPs are clearly capable of redistributing in all directions away from the cisterna magna. The distribution of FNPs was symmetrical between the hemispheres, with both sagittal and coronal brain sections demonstrating that FNPs concentrate along the ventral surfaces of the brain. Lesser delivery of FNPs to the dorsal surface of the brain was observed here. This phenomenon does not appear to be dependent on the size of the infused agent, as it was similarly observed for both a small molecule (99mTc-DMSA, ~500 Da) and a protein (idursulfase, 76 kDa) following lumbar injection in healthy rats^14,15^, as well as for 10 µm MPs in this work. A relatively high FNP concentration was observed in the supracerebellar cistern at all time points, although a relatively small population of FNPs moved past the cistern towards the olfactory bulb. We saw a similarly pronounced concentration of FNPs within the pituitary recess on the ventral side of the brain, although significantly more FNPs moved rostrally beyond the recess. These data suggest there must be inherent differences in net movement/clearance of CSF or features of the trabecular mesh along the dorsal versus ventral surfaces and microanatomy of the brain. To our knowledge, this has not been directly observed in prior publications.

Although FNPs were capable of accessing all leptomeningeal surfaces, the concentration of FNPs was not uniform with respect to brain and spinal cord anatomy. This study does not assess whether the distinct patterns of delivery observed for FNPs could be generalized to other substances injected into the CSF, such as small molecules, proteins, or gene therapies. However, the relative scale of differences observed for FNP delivery were of a magnitude that would be expected to influence the activity of therapeutic molecules. These results highlight the need for additional work to further elucidate how microanatomy of the leptomeninges and regional CSF dynamics influence the distribution and activity of molecules delivered via the IT route.

CSF is continually produced by the choroid plexus and turns over multiple times a day (4×/day in human, 12×/day in mice)^42,43^. The half-life of IT administered molecules is typically on the order of a few hours^17^, and clearance remains a significant obstacle to effective drug delivery. CSF clearance is proposed to occur through multiple routes, including direct transfer to blood through arachnoid villi, movement across the cribriform plate into the nasal mucosa, and glymphatic clearance from perineural transport along both cranial and spinal nerve roots. The relative significance of each of these routes is often debated and varies by species^31,42,44–47^. Here, we found that nondegradable, FNPs remained distributed throughout the murine neuraxis at a relatively high concentration for over 3 weeks. Both IVIS and confocal data suggest the majority of FNP clearance occurs from the brain compartment, with minimal clearance along the spinal cord. In agreement with prior works in rodents, we saw FNP movement across the cribriform plate into the nasal mucosa at all time points. One possible explanation for the lack of FNPs along the dorsal brain could be clearance of FNP into venous circulation through arachnoid villi along the Superior Sagittal Sinus (SSS). However, recent works suggest direct CSF to blood clearance is minimal in mice^45,46^, and we failed to observe direct interactions of the FNPs within the SSS in coronal sections. Additionally, the lack of FNP signal within the liver suggests minimal to no clearance of FNP to the blood. Future studies using whole body imaging would be necessary to fully understand the contribution of each of these clearance routes in the context of NP distribution.

NP size is one of the most important biophysical parameters governing fate of NPs following their administration by various routes, including intravenous^48,49^, oral^50,51^, intranasal^52,53^, or convection enhanced delivery^54,55^. DepoCyt is a 10 µm multilamellar liposome that has been shown to reach lumbar spinal cord within a day following intraventricular administration in humans^56^. These data suggest that large particles are capable of navigating the SAS to reach distal regions of the CNS. However, because trabeculae form a porous mesh between the arachnoid mater and pia mater, we expected that the distribution of larger sized MPs would be restricted relative to smaller sized NPs, particularly within the smaller anatomy of the mouse^57,58^. In contrast to this expectation, we found MPs were capable of navigating the SAS to the sacral spinal cord within 2 hours of IT administration in mice, although their distribution was not as complete as smaller sized FNPs. Most distal slices contained no MPs and those that did never contained more than 1 MP/slice. Thus, while microparticle systems can help overcome the issue of rapid drug clearance through prolonged drug release, the minimal number of MPs in distal regions suggests that these systems must inevitably rely on movement of free drug from the particle through CSF to reach its target. Smaller NP systems, such as the FNPs used here, could overcome this delivery barrier to deposit drug more uniformly within the SAS. In addition, their small size means they can more easily interact with or be more easily taken up into cells, allowing for opportunities to increase delivery to specific cells or tissues through active targeting. The minimal penetration of 100 nm NPs across the pia mater or diffusion along perivascular routes in the parenchyma likely limits their use for applications requiring delivery to deep CNS regions. Although NPs as large as 100 nm can maneuver through brain perivascular spaces under convection enhanced delivery^54^, sub-50 nm and ideally >20 nm NPs are likely necessary to achieve significant penetration from the SAS into the brain^59,60^.

Ultimately, our data provide the first direct evidence that 100 nm NPs are capable of rapidly distributing through the SAS of the brain and spinal column following IT administration in healthy mice. The retention of 100 nm FNPs within the leptomeningeal space supports future work examining their potential for overcoming the pharmacokinetic limitations of therapies and imaging agents for treating and tracking diseases of the meninges, including meningitis, meningiomas and leptomeningeal metastasis from solid tumors^61,62^. Controlled release properties of nanocarriers could be utilized to provide prolonged drug exposure while reducing healthy tissue exposure to high peak drug concentrations. Although the non-biodegradable NPs used here were retained for over 3 weeks, many NPs used for drug delivery are degradable; thus, overall retention and efficacy of a nanomedicine could be dictated by both carrier degradation and drug release rates. IT administration has the further advantage of exposing nanoparticles more directly to target tissues and cells within the SAS, which could open new possibilities for targeted drug delivery and imaging. Taken in sum, these studies provide foundational characterization of the fate of nanoscale colloids following IT injection in healthy mice. This work opens new opportunities and potential challenges to consider for the delivery of drugs from NPs administered directly to the CSF to treat disease.

## Materials and Methods

### Materials

100 nm carboxylate-modified FluoSpheres^TM^ (red fluorescent, Ex/Em 580/605 nm), 10 µm FluoSpheres (green fluorescent, Ex/Em 468/508 nm) and 20x Borate Buffer were purchased from ThermoFisher Scientific (Waltham, MA USA). Endotoxin-free water was obtained from G-Biosciences (St. Louis, MO, USA). Poly(ethylene glycol)-amine (mPEG-Amine, 2 kDa MW) was purchased from Creative PEGWorks (Chapel Hill, NC USA). Other chemicals or reagents were purchased from Sigma-Aldrich (St. Louis, MO USA) unless specified.

### FluoSphere PEGylation and characterization

Carboxylate-modified FluoSphere^TM^ NPs (FNP, 100 nm) were covalently modified with mPEG-Amine (2 kDa MW) by carbodiimide chemistry as previously described by Nance *et al*.^54^. FNPs were washed to remove sodium azide using 0.5 ml Amicon-Ultra centrifugation filters (10k MWCO). mPEG-amine (5x molar excess) was added and allowed to stir for 15 min. Next, 6.5 mg N-Hydroxysuccinimide (NHS) dissolved in 6 ml borate buffer (200 mM, pH8) was added, followed by 15.4 mg 1-Ethyl-3-(3-dimethylaminopropyl) carbodiimide (EDC). After 3 hours, the reaction was quenched with excess glycine (100 mM) for 30 min. Unreacted PEG, EDC, NHS and glycine were removed by dialysis (100k MWCO) for 24 hours. FNPs were washed and collected by centrifugation using Amicon-Ultra centrifugation filters (100k MWCO) and resuspended to 20 mg/ml in 1x PBS for storage at 4 °C.

FNP size and zeta potential was measured in 1 mM KCl before and after PEGylation using a NanoBrook 90Plus Zeta (Brookhaven Instruments, Holtsville, NY USA). Pegylated FS stability in 10% FBS at 37 °C was measure at 1, 2, 4, 12, 24 hours and 1 week. 1 mg of lyophilized sample was dissolved in 0.6 mL of chloroform-d (CDCl3). H-NMR spectra of the samples were obtained using a 300 MHz Bruker Avance NMR spectrometer at standard room temperature.

### *In Vivo* studies

All experiments, procedures and animal care practices were approved by the Barrow Neurological Institute’s Institutional Animal Care and Use Committee and were performed in accordance with all relevant guidelines. Healthy, female C57 albino mice (Charles River) were used for *in vivo* experiments.

### FNP Administration

FNPs were administered directly into the CSF of the cisterna magna through the atlanto-occipital membrane between the skull and C1 vertebrae. Mice were anesthetized with ketamine/xylazine (100/10 mg/kg) and mounted in a stereotaxic frame (Kopf Instruments, Tujunga, CA, USA). A Hamilton syringe (29 gauge needle, 30° beveled tip) was used to inject 2 µl FNPs over 1 min at a depth of 1 mm, and the needle was left in place for an additional 2 min before removing. All animals received a subcutaneous (SQ) injection of Buprenorphine SR (1 mg/kg) prior to surgery, and ibuprofen was provided in their water for 1 week to control pain.

### *In Vivo* FNP Tracking

Macroscopic images of FNP distribution and semi-quantitative measurements of clearance kinetics (5 min, 2, 6, 24 and 48 hours after injection) were obtained using a Xenogen IVIS Spectrum *In Vivo* Imaging System (Ex 570/Em 620). Fur was removed from the neck and back prior to imaging. A line profile was drawn along the length of the neuraxis to quantify intensity. Background signal measured from a pre-injection image of each subject was subtracted from each time point. FNP intensity at each time point was normalized within each subject to the maximum measured intensity at 5 min.

### *Ex Vivo* neuraxis collection

Mice were perfused transcardially 2, 6, 24 and 48 hours, or 1 and 3 weeks post FNP administration with PBS followed by 4% paraformaldehyde (PFA). The entire neuraxis from nasal sinuses to sacral spinal region was isolated intact, leaving the bone in place, and post-fixed in 4% PFA for an additional 48 hrs. The bone was decalcified over 5 days in a 4% HCl and 4% Formic Acid (v/v) solution. After cryoprotection in a 30% (w/v) sucrose solution for 48 hrs, the brain and spinal cord were cut at the C1 vertebrae to enable separate processing.

### Brain and spinal column processing

OCT embedded spinal cord (coronal) and brain (sagittal) were sliced to a thickness of 20 µm and counterstained with Vectashield plus DAPI. Slides were imaged (FNPs: 560/605 nm, MPs: 468/508 nm) on an inverted confocal microscope. Laser intensities and gain were maintained across samples and time points. For quantification of the spinal cord distribution, an average background pixel intensity (non-injected controls) was subtracted from each image and the FNP intensity was measured by ImageJ (v1.47, NIH).

### Statistical testing

All statistical tests were performed using GraphPad Prism 5. Comparisons of IVIS brain and spinal cord AUC over time were made with a 1-way ANOVA with Tukey’s Multiple comparison post hoc test. A 1-way ANOVA with Bonferroni multiple comparisons test was used to compare area under the curve (AUC) of spinal cord curve fits, expressed as arbitrary units (AU) * time (hrs). Statistical significance is reported for p < 0.05.

## Supplementary information

Supplemental Information
