# Supplementary material for: Fate of nanoparticles in the central nervous system after intrathecal injection in healthy mice
Source: Sci Rep. 2019 Aug 29;9:12587. doi: 10.1038/s41598-019-49028-w (PMC6715675; doi:10.1038/s41598-019-49028-w)
Supplement: Supplementary file 1 — Supplemental Information [file 41598_2019_49028_MOESM1_ESM.docx]

Supplemental Information

Fate of nanoparticles in the central nervous system after intrathecal injection in healthy mice

Householder KT^1-3^, Dharmaraj S^1-3^, Sandberg DI^1,4^, Wechsler-Reya RJ^5^, Sirianni RW^1-3 *^

^1^ Vivian L. Smith Department of Neurosurgery, McGovern Medical School, University of Texas Health Science Center at Houston, 6431 Fannin St, Houston, TX, 77030

^2^ Barrow Brain Tumor Research Center, Barrow Neurological Institute, 350 W. Thomas Rd, Phoenix, AZ, 85013, USA

^3^ School of Biological and Health Systems Engineering, Ira A. Fulton Schools of Engineering, Arizona State University, P.O Box 879709, Tempe, AZ, 85287, USA

^4^ Departments of Pediatric Surgery and Neurosurgery, McGovern Medical School, University of Texas Health Science Center at Houston, 6431 Fannin St, MSB 5.144, Houston, TX, 77030

^5^ NCI-Designated Cancer Center, Sanford-Burnham Medical Research Institute, 10901 N Torrey Pines Rd, La Jolla, CA, 92037, USA


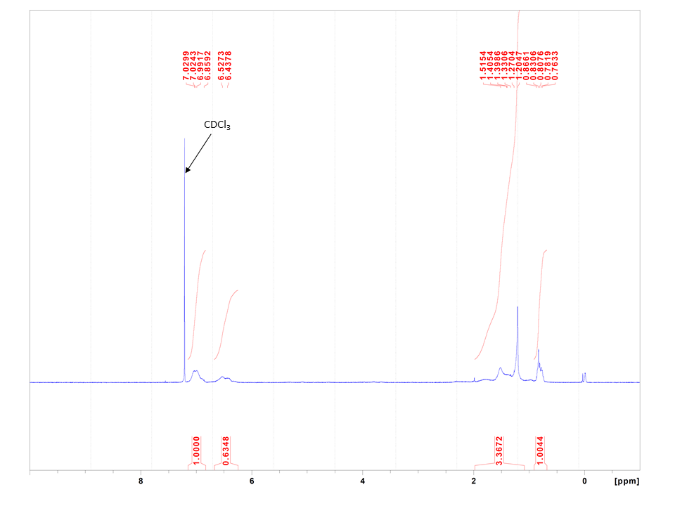

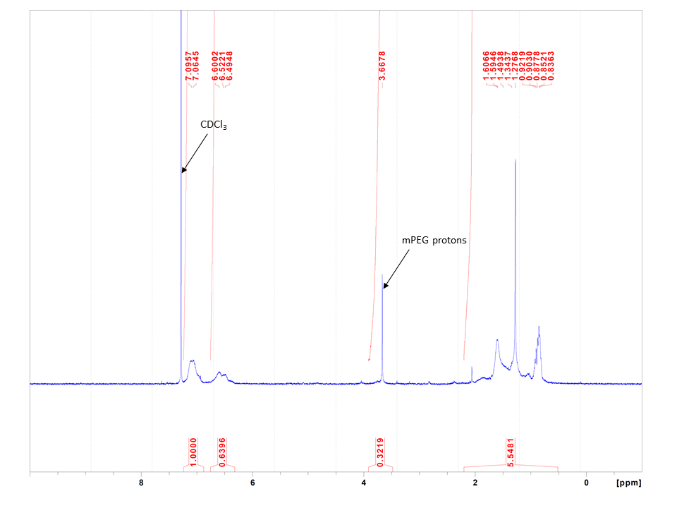


Figure S1: ^1^HNMR plots for pegylated (right) and non-pegylated FS (left). The appearance of a peak at 3.6 in the pegylated FS plot confirms successful attachment of PEG to the FS surface.


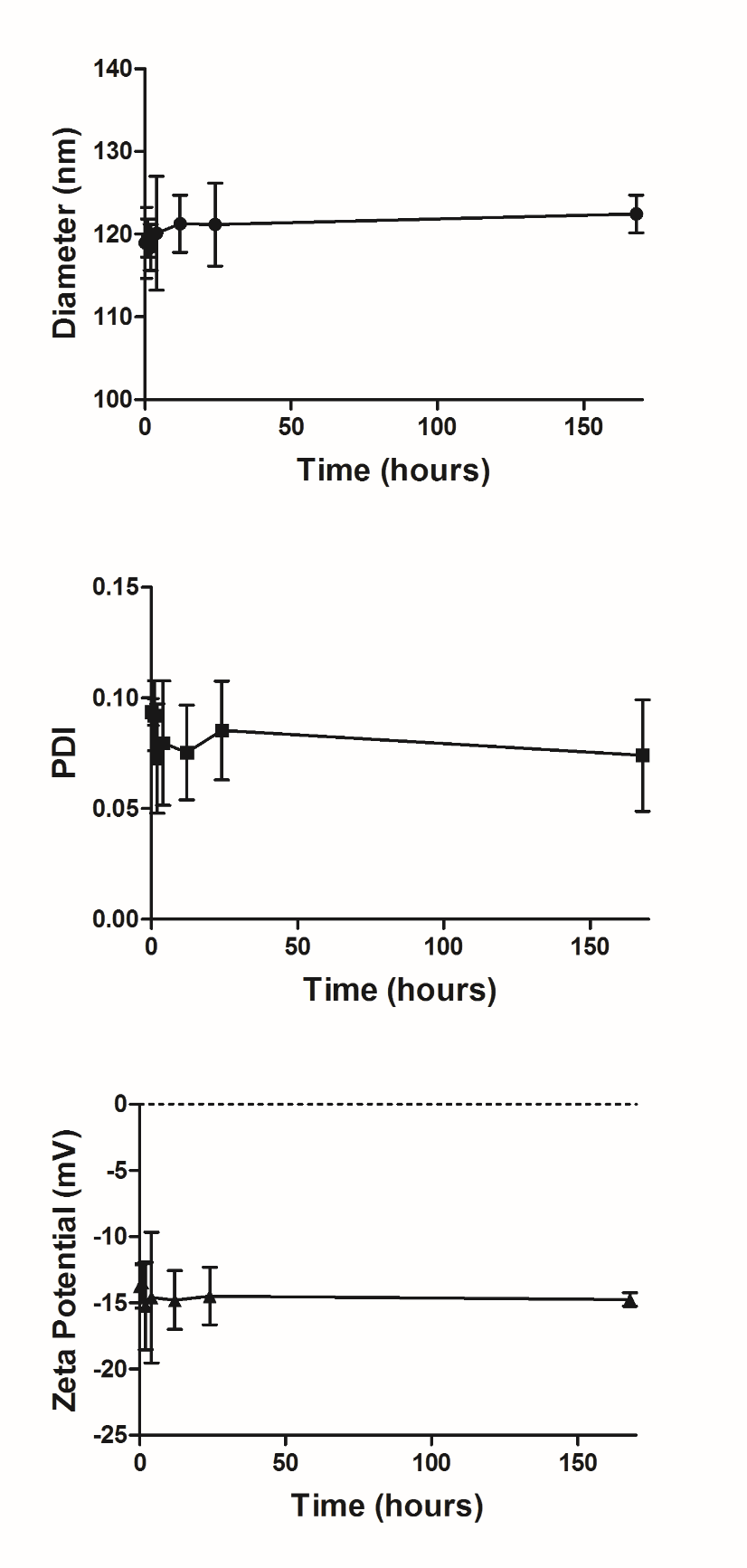


Figure S2: FNP stability in 10% FBS. FNPs incubated in 10% FBS at 37°C maintained consistent size, PDI and zeta potential for over 1 week. Mean ± SD


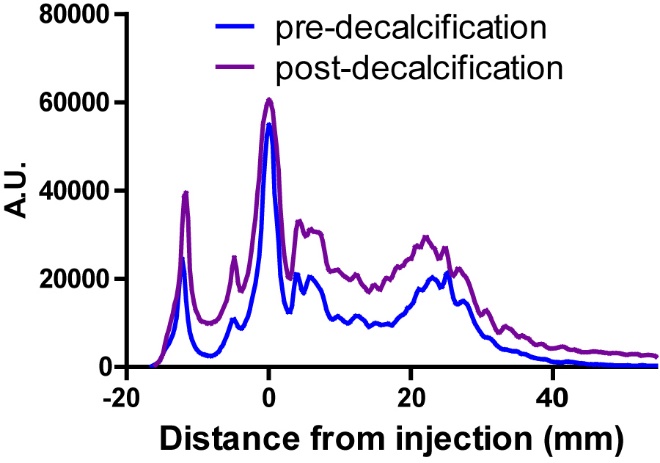


Figure S3: FNP intensity measured by IVIS along the neuraxis before and after decalcification. An increase in FNP signal was observed, likely from decreased attenuation from decalcified bone. Decalcification was confirmed to not alter the spatial distribution of the FNPs.
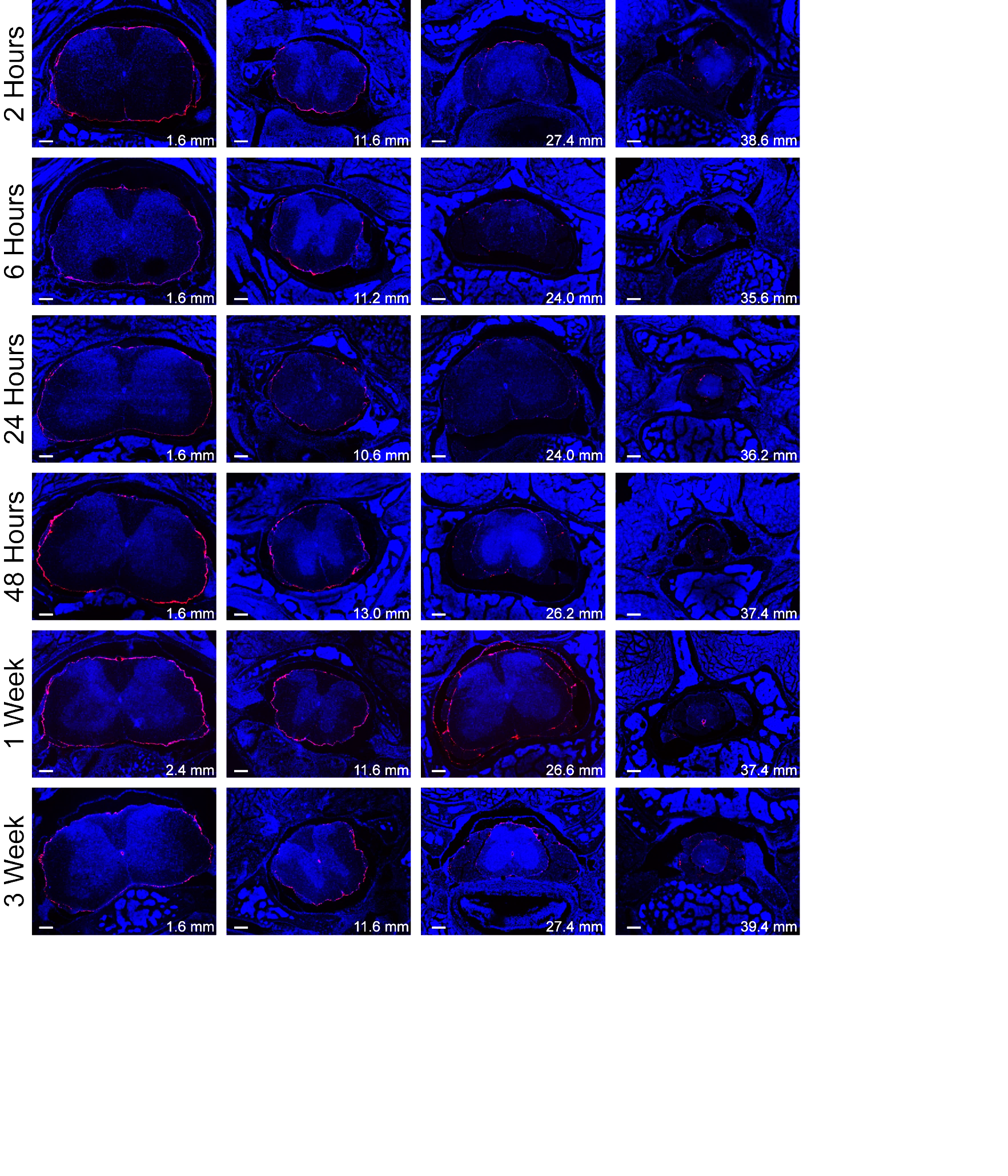


Figure S4: Representative confocal images of FNP distribution at different locations along the spinal column over time. FNP (red) delivery along the spinal column appeared to be consistent over time. Equivalent linear adjustments were made to the FNP signal to enable better visualization. Cell nuclei (DAPI) are shown in blue. Scale bar = 200 µm.


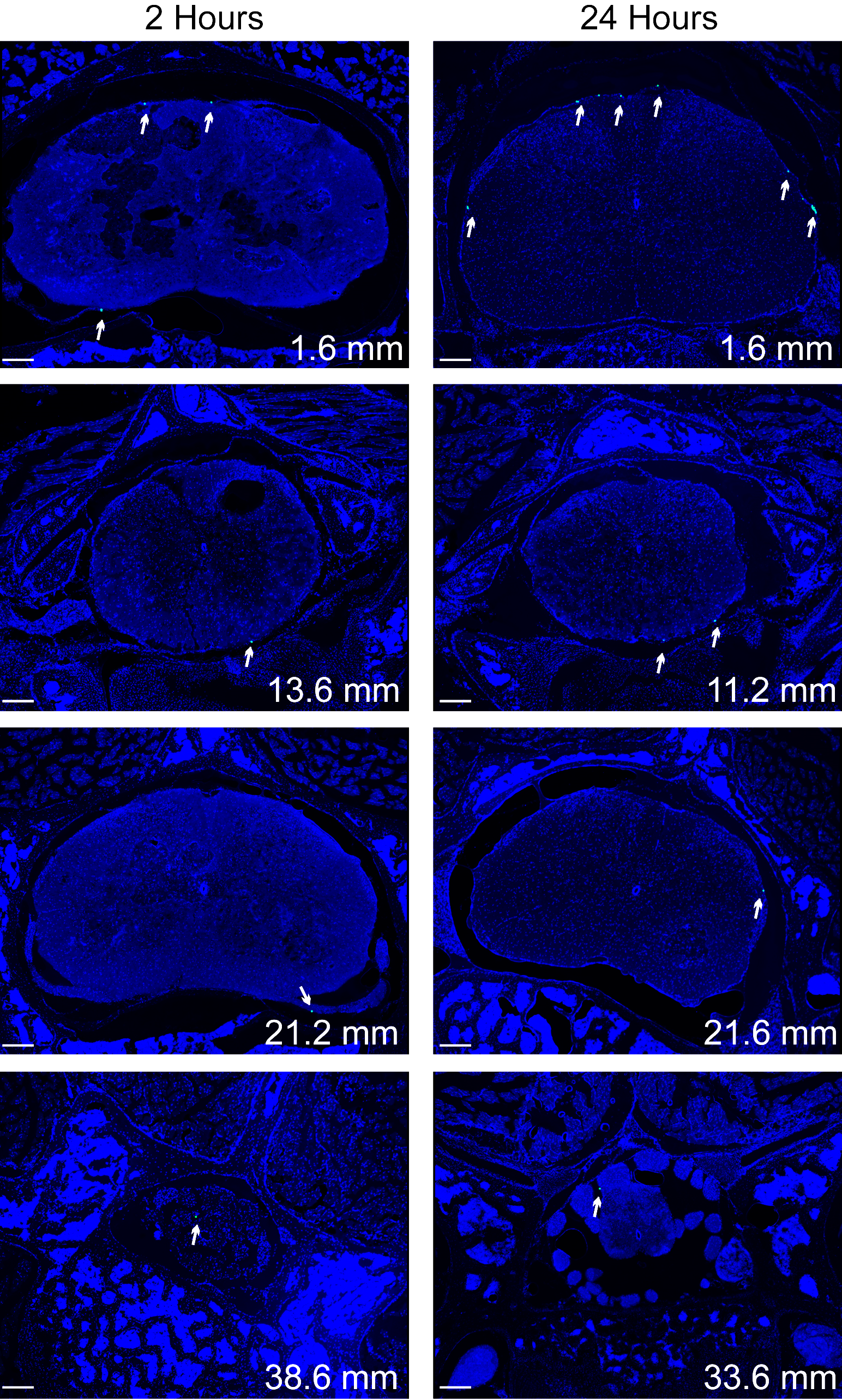


Figure S5: Representative confocal images of MP distribution at different locations along the spinal column. MPs (green, arrows) were able to transverse the length of the spinal column to the sacral spinal cord within 2 hours, albeit very infrequently. Cell nuclei (DAPI) are shown in blue. Scale bar = 200 µm.


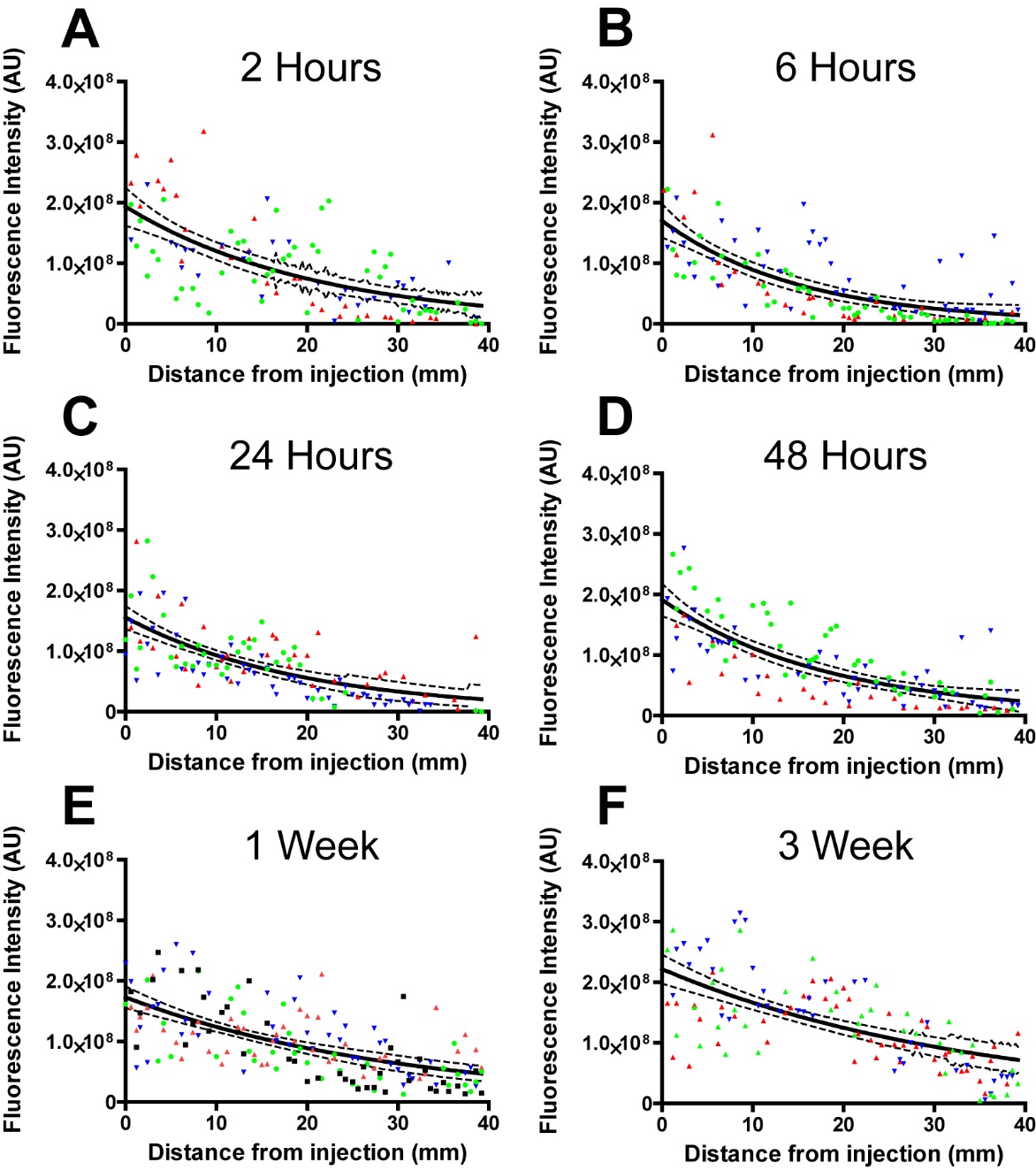


Figure S6: Scatter plots for each time point show the distribution of measured FNP intensity and the resulting exponential curve fit of all the data points (solid line) with the 95% confidence interval (dashed line). Each point represents the measured intensity within a given tissue slice. Within each graph, the same color and shape represent tissue slices from the same animal.


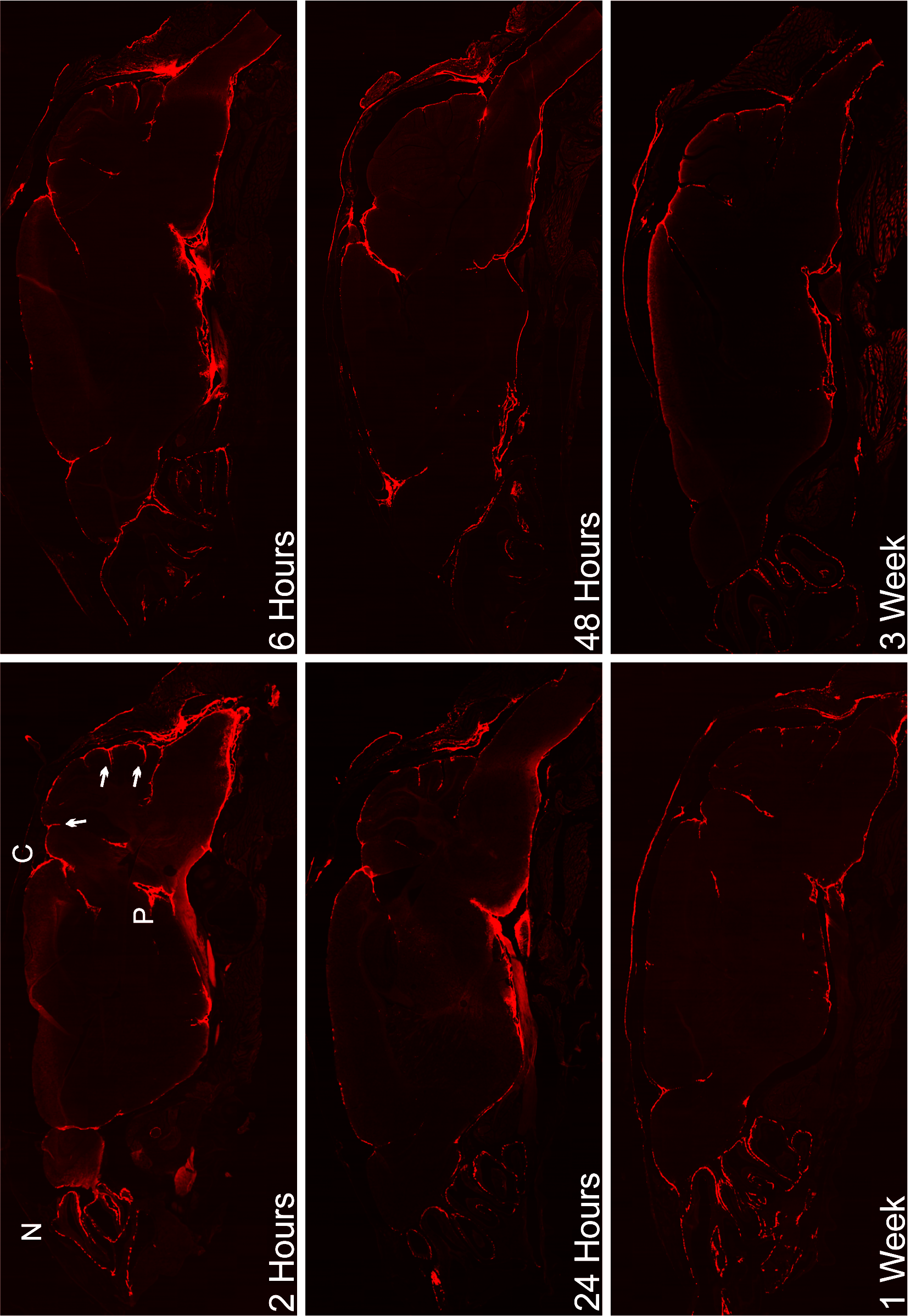


Figure S7: Representative confocal images of FNP distribution around the brain. FNPs (red) were distributed around the entire brain and could be seen following the meninges into the sulci of the cerebellum (arrows). There was a strong preference for ventral distribution and consistently high delivery to the supracerebellar cistern (C) and pituitary recess (P). Clearance of FNPs across the cribriform plate into the nasal mucosa (N) was seen at all time points. Qualitatively, a general decrease in total FNP intensity was observed over time, consistent with IVIS data. Linear corrections were applied equally across images to better show FNP distribution. Scale bar = 500 µm.


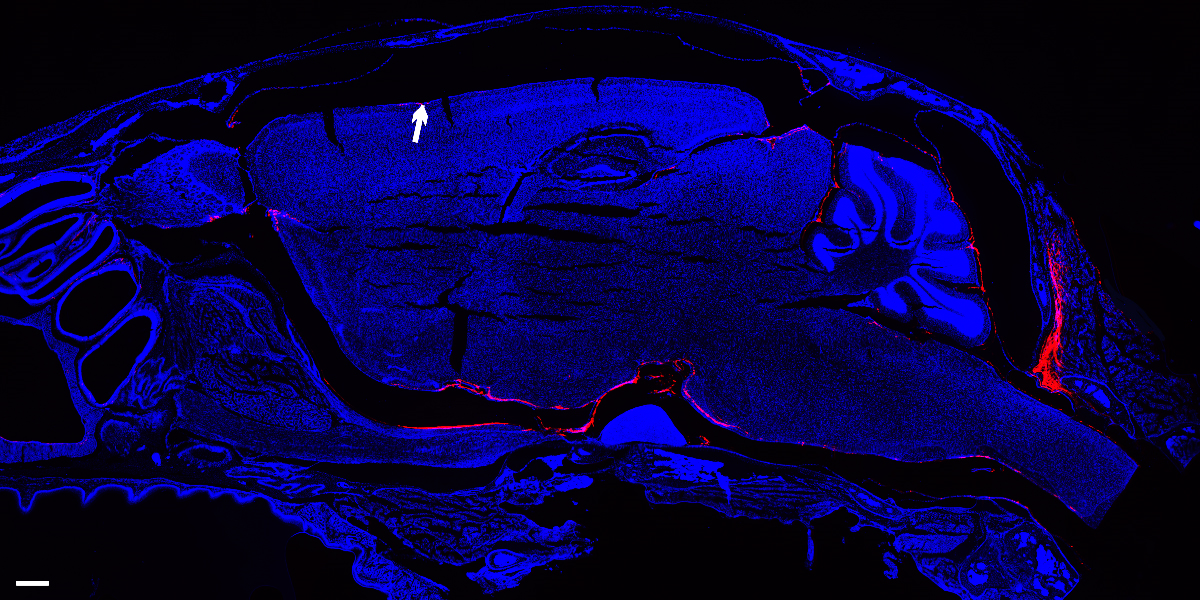


Figure S8: Mouse position does not affect FNP distribution around the brain. Brain image from a mouse allowed to recover on its back during the 2 hours after injection. FNP (red) distribution was still favored towards the ventral brain and minimal delivery was observed to the prefrontal cortex regions of the SAS (arrow). Cell nuclei are shown in blue. Scale bar = 500 µm.
